# QTL Analysis of Kernel-Related Traits in Maize Using an Immortalized F_2_ Population

**DOI:** 10.1371/journal.pone.0089645

**Published:** 2014-02-28

**Authors:** Zhanhui Zhang, Zonghua Liu, Yanmin Hu, Weihua Li, Zhiyuan Fu, Dong Ding, Haochuan Li, Mengmeng Qiao, Jihua Tang

**Affiliations:** 1 College of Agronomy/Key Laboratory of Physiological Ecology and Genetic Improvement of Food Crops in Henan Province, Henan Agricultural University, Zhengzhou, China; 2 Department of Biological Sciences, Michigan Technological University, Houghton, Michigan, United States of America; Kansas State University, United States of America

## Abstract

Kernel size and weight are important determinants of grain yield in maize. In this study, multivariate conditional and unconditional quantitative trait loci (QTL), and digenic epistatic analyses were utilized in order to elucidate the genetic basis for these kernel-related traits. Five kernel-related traits, including kernel weight (KW), volume (KV), length (KL), thickness (KT), and width (KWI), were collected from an immortalized F_2_ (IF_2_) maize population comprising of 243 crosses performed at two separate locations over a span of two years. A total of 54 unconditional main QTL for these five kernel-related traits were identified, many of which were clustered in chromosomal bins 6.04–6.06, 7.02–7.03, and 10.06–10.07. In addition, *qKL3*, *qKWI6*, *qKV10a*, *qKV10b*, *qKW10a*, and *qKW7a* were detected across multiple environments. Sixteen main QTL were identified for KW conditioned on the other four kernel traits (KL, KWI, KT, and KV). Thirteen main QTL were identified for KV conditioned on three kernel-shape traits. Conditional mapping analysis revealed that KWI and KV had the strongest influence on KW at the individual QTL level, followed by KT, and then KL; KV was mostly strongly influenced by KT, followed by KWI, and was least impacted by KL. Digenic epistatic analysis identified 18 digenic interactions involving 34 loci over the entire genome. However, only a small proportion of them were identical to the main QTL we detected. Additionally, conditional digenic epistatic analysis revealed that the digenic epistasis for KW and KV were entirely determined by their constituent traits. The main QTL identified in this study for determining kernel-related traits with high broad-sense heritability may play important roles during kernel development. Furthermore, digenic interactions were shown to exert relatively large effects on KL (the highest AA and DD effects were 4.6% and 6.7%, respectively) and KT (the highest AA effects were 4.3%).

## Introduction

Many biologically and agriculturally important traits are defined by complex genetic mechanisms. They are controlled by interacting networks comprised of multiple genes with relatively small genetic effects, and determined by their constituent traits [1], [2]. Maize kernel weight is a typical quantitative trait that is controlled by multiple genes and environmental factors [3]–[6], and can be dissected into several secondary components, including kernel density, volume, length, width, and thickness [7], [8]. Maize kernel weight is also affected by multiple biological processes. These processes can be studied at different organizational levels, particularly with respects to two very important maize yield-related traits: kernel size and kernel growth rate [9]–[11]. In maize breeding programs, kernel size is an important breeding target both because of end-use quality requirements and consumer preference, as well as the fact that it is a grain yield component [12]. Kernel development is divided into three phases: lag, effective-filling, and maturation drying stages [3], [4], [9]. During the kernel development process, kernel growth rate is dynamic and determines the final kernel weight [4]. Numerous studies have focused on kernel development at the cellular and tissue level [4], [13], [14], as well as kernel growth at the whole-plant level during the grain-filling period [9], [15], [16].

Kernel-related traits are classic quantitative traits regulated by multiple quantitative trait loci (QTL) and gene interactions at the various kernel developmental stages. To elucidate the genetic basis of kernel-related traits in maize, many QTL for kernel weight (e.g., 100- and 300-kernel weight)—a primary grain yield determinant—have been identified over the last two decades [17]–[22]. In contrast, only a few QTL have been identified for kernel weight secondary traits, including kernel volume, length, width, and thickness [7], [8], [23]. Recently, several qualitative genes for kernel size and weight have also been isolated by making use of maize mutants, *rgf1* [24], *sh1* and *sh2* [25], *dek1* [26], and *incw2* [4], [27], [28]. The genetic architecture of maize kernel weight and size, however, has not been completely elucidated, and the genetic relationships between kernel weight and size to their secondary traits are not fully understood.

To investigate the genetic relationships between kernel weight and its secondary component traits, we used a statistical procedure for analyzing conditional genetic effects [2] in combination with QTL mapping [29]. Here, the KW was separated into KL, KT, KWI, and KV components. For example, if KW is genetically correlated with KT, conditioning KW on KT allows for the dissection of KW independently of variation in KT. Using this methodology, the KW conditional values based on its secondary traits can then be analyzed by QTL mapping. By comparing unconditional and conditional QTL for KW, genetic relationships between KW and KT or other kernel-related traits can be identified at the individual QTL level. Consequently, the genetic relationship between KW and KT has four possible results: (1) a QTL for KW identified by unconditional QTL mapping has a similar or equal effect, meaning that the QTL is expressed independently of KT; (2) a QTL detected by the unconditional method shows a greatly reduced or enhanced effect, indicating that this QTL for KW is partially associated with KT; (3) a QTL is identified only by unconditional QTL mapping, meaning that this QTL for KW is entirely depended on KT; or (4) a QTL is only detected by conditional mapping, indicating that the QTL for KW is completely suppressed by KT [30]–[32]. The results of such an analysis can provide valuable information for improving maize grain yield and quality via marker-assisted selection. This method has been used successfully in identifying genetic relationships between oil content and its related/causal traits in rapeseed [33], plant height and lengths of the spike and internode in wheat [30], kernel weight per spike and its components in wheat [31], and kernel weight and kernel dimensions in wheat [32].

Because hybrid maize is widely grown throughout the world, studies of hybrid populations are both agronomically and economically important. In maize hybrids, grain yield and its associated traits are controlled by additive and/or dominant QTL and digenic interaction effects. IF_2_ populations, which are composed of different crosses derived from recombinant inbred lines (RILs) and/or doubled haploid (DH) populations [34], can be used to detect the additive and dominant effects of QTL mapping. In addition, compared with RILs and DH populations, IF_2_ populations are as genetically as informative as an F_2_ population, and have an identical genetic background. IF_2_ populations are therefore ideal systems for dissecting the genetic basis of grain yield and its components in maize, and the resulting information can be directly used for maize breeding. Dissecting the genetic basis of these kernel parameters using an IF_2_ population can contribute to our understanding of kernel architecture and help improve kernel quality. The present study, which isolated KW into several secondary constituent traits, aimed to: (1) elucidate QTL for kernel development-related traits using an immortalized F_2_ population derived from pairwise intercrossing of the 166 recombinant inbred lines (RILs) (Nongda 108, Huang C × Xu 178); (2) evaluate the genetic influence of variation in various kernel secondary traits on kernel size and KW; and (3) detect digenic epistatic effects for kernel-related traits.

## Results

### Phenotypic variation in kernel-related traits

The five studied kernel-related traits—KL, KWI, KT, KV, and KW—showed high broad-sense heritabilities of 86.5%, 90.2%, 89.4%, 70.5%, and 87.0%, respectively (Table 1). In both years, the Nongda 108 hybrid had higher KW and KL values than those of its parents (Huang C and Xu 178). In contrast, KWI and KT were smaller for the hybrid than for its parents, and the KV value of the hybrid was lower than in the Huang C parent but higher than in the Xu 178 parent over the two years of the study. When the IF_2_ population was compared with the hybrid, the average values of the five measured kernel-related traits were found to be smaller in the IF_2_ population than in the Nongda 108 hybrid. In contrast, the maximum values for the IF_2_ population in both 2009 and 2010 were higher than in the Nongda 108 hybrid, indicating that there was non-optimal heterosis of these kernel-related traits in the Nongda 108 hybrid. Comparing the IF_2_ population to the parents, the KL value of the IF_2_ population was higher in 2009 and 2010 at both experimental locations, whereas KW, KT, and KWI were smaller. Within the four environments, the five measured kernel-related traits in the IF_2_ population displayed significant differences (*p*<0.05). Both KW and KV exhibited extremely significant positive relationships with each other, and had significant positive relationships with KWI and KT, yet no significant relationship with KL (Table 2). For the other three kernel characteristics, only KWI positively correlated significantly with KT.

**Table 1:** Performance of kernel-related traits in the immortalized F_2_ population.

Year	Location	Trait ^a^	F_1_	Parents		IF_2_		
				Huang C	Xu 178	Mean ± SE	Range	CV(%)^b^
2009	Zhengzhou	KL	1.03	0.72	0.63	0.88±0.005	0.62–1.06	7.74
		KWI	0.66	0.80	0.69	0.65±0.003	0.51–0.79	7.55
		KT	0.46	0.64	0.56	0.44±0.002	0.38–0.59	5.60
		KV	0.24	0.26	0.20	0.21±0.002	0.15–0.28	10.74
		KW	0.27	0.26	0.26	0.24±0.002	0.18–0.36	12.15
	Anyang	KL	0.98	0.89	0.75	0.88±0.005	0.65–1.07	7.61
		KWI	0.68	0.75	0.68	0.65±0.003	0.52–0.79	7.29
		KT	0.48	0.63	0.56	0.44±0.002	0.39–0.51	5.23
		KV	0.25	0.27	0.20	0.20±0.002	0.14–0.27	10.91
		KW	0.29	0.27	0.23	0.24±0.002	0.17–0.32	12.04
2010	Zhengzhou	KL	0.91	0.69	0.53	0.86±0.005	0.60–1.03	8.51
		KWI	0.71	0.74	0.76	0.64±0.003	0.51–0.77	7.52
		KT	0.44	0.64	0.52	0.44±0.002	0.38–0.50	5.66
		KV	0.24	0.26	0.22	0.20±0.002	0.14–0.29	11.73
		KW	0.28	0.25	0.25	0.24±0.002	0.17–0.32	11.39
	Anyang	KL	0.98	0.69	0.64	0.88±0.005	0.64–1.09	7.59
		KWI	0.69	0.74	0.7	0.64±0.003	0.52–0.76	7.53
		KT	0.42	0.64	0.55	0.44±0.002	0.39–0.51	5.53
		KV	0.25	0.26	0.21	0.20±0.002	0.15–0.29	10.75
		KW	0.29	0.25	0.25	0.24±0.002	0.16–0.34	12.19
	IF_2_		KL	KWI	KT	KV	KW	
		*h^2^b*(%)^c^	86.5	90.2	89.4	70.5	87.0	
		*p* value^d^	1.08E-13	2.68E-04	2.01E-7	0.021	0.001	

Notes: ^a^ KL, kernel length; KWI, kernel width; KT, kernel thickness; KV, kernel volume; KW, kernel weight;

b CV, coefficient of variation;

c *h^2^b*, broad-sense heritability;

d *p* value, statistical significance of kernel-related traits in the four environments.

**Table 2:** Correlation coefficients among five kernel-related traits in the immortalized F_2_ population.

Location		KL	KWI	KT	KV	KW
Zhengzhou	KL		−0.02	−0.19	0.13	0.10
	KWI	0.12		0.31^**^	0.55^**^	0.61^**^
	KT	−0.17	0.30^**^		0.35^**^	0.47^**^
	KV	0.13	0.67^**^	0.41^**^		0.72^**^
	KW	0.15	0.64^**^	0.48^**^	0.72^**^	
Anyang	KL		0.13	−0.04	0.25^*^	0.18
	KWI	−0.04		0.30^**^	0.64^**^	0.72^**^
	KT	−0.12	0.27^**^		0.35^**^	0.52^**^
	KV	0.05	0.59^**^	0.36^**^		0.72^**^
	KW	−0.04	0.68^**^	0.47^**^	0.79^**^	

Notes: ^**^ Significant correlation (*p*≤0.01).

Correlation coefficients for 2009 are above the diagonal, while those for 2010 are below the diagonal.

### Unconditional QTL for kernel-related traits were detected in the IF_2_ population

**Figure 1:**
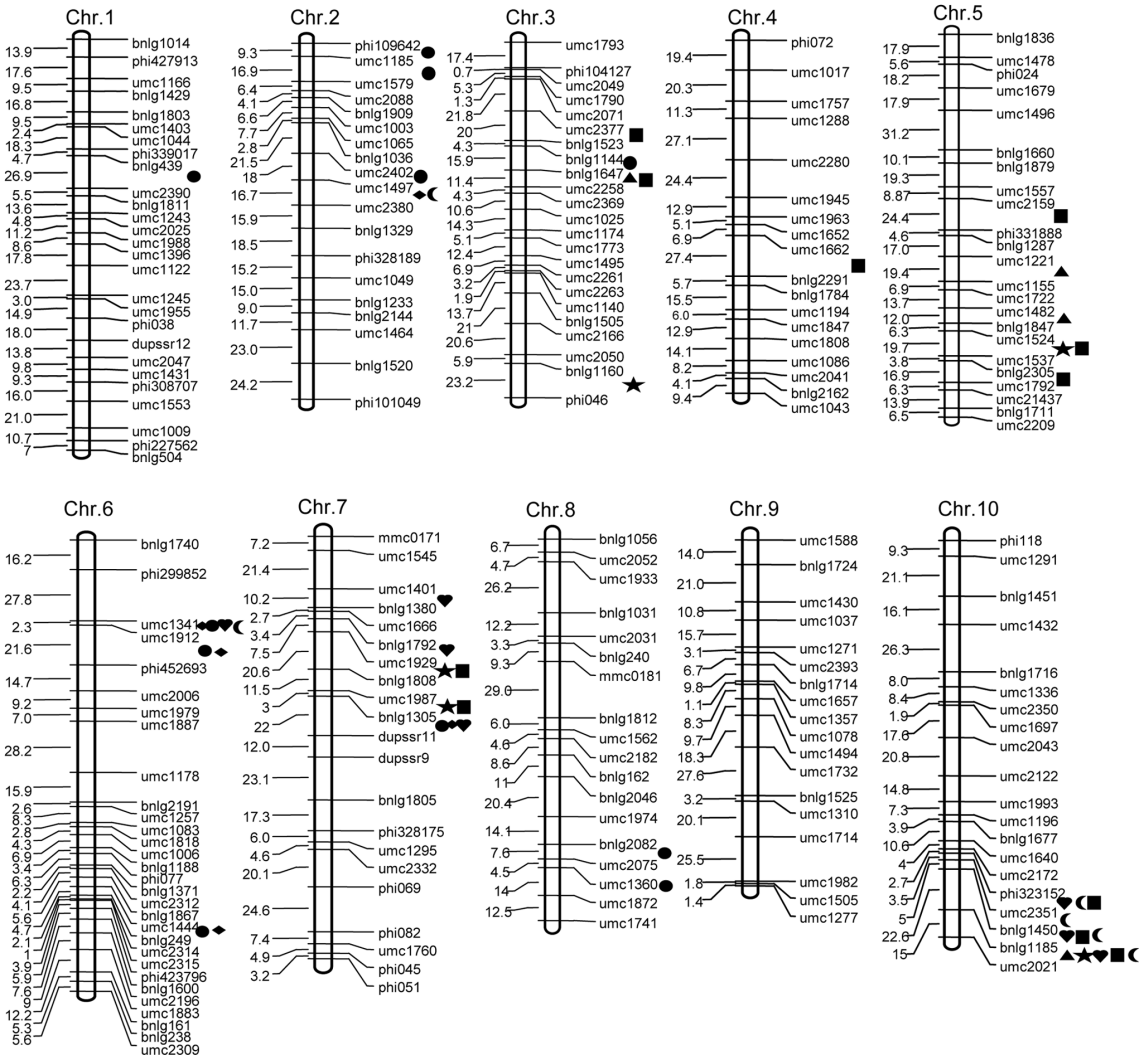
Chromosomal locations of QTL for kernel-related traits detected in the immortalized F_2_ maize population. Note: *Triangle*, unconditional QTL for kernel length; *Rhombus*, unconditional QTL for kernel width; *Heart*, unconditional QTL for kernel volume; *Star*, unconditional QTL for kernel weight; *Moon*, conditional QTL for kernel volume; and *Square*, conditional QTL for kernel weight.

A total of 42 main-effect QTL were detected based on the averaged data for each IF_2_ line (derived from three replicates per environment). These QTL were distributed across all chromosomes with the exception of chromosomes 4 and 8 (Table 3; Fig. 1). Five QTL for KL were identified in the four environments, with one of them, *qKL3*, contributing 11.2% and 16.2% of the total phenotypic variance at the Anyang site during 2009 and 2010, respectively. Of the eight QTL for KWI detected over the four environments, *qKWI6a* contributed 17.4%, 21.1%, 17.1%, and 18.4% of the total variance in the four environments. Seven QTL for KT, accounting for 6.1–21.1% of the total phenotypic variance, were identified on chromosomes 1, 2, 6, and 8. For KV, 11 QTL were detected in the four environments. *qKV10a*, which was identified at both locations over the two years, was responsible for 22.3%, 26.3%, 23.8%, and 24.9% of the total phenotypic variance. In addition, *qKV10b* was detected at both locations in 2010, and accounted for 25.6% and 22.3% of the total variance. A total of 11 QTL for KW were detected in the IF_2_ population. *qKW10a* was detected at Zhengzhou and Anyang in 2009 and at Anyang in 2010, and contributed 14.9%, 16.6%, and 13.5% of the total phenotypic variance, respectively. Finally, *qKW7a* was detected at Zhengzhou in 2009 and at Zhengzhou and Anyang in 2010, and was responsible for 10.6%, 12.7%, and 10.5% of the total variance, respectively.

**Table 3:** Unconditional QTL detected for kernel-related traits in the immortalized F_2_ population.

Year	Location	Trait^a^	QTL^b^	Markers interval	LOD^c^	A^d^	D^d^	Effects^e^	R^2^ (%)^f^
2009	Zhengzhou	KL	*qKL5a*	*umc1221-umc1155*	5.9	−0.042	0.012	PD	*14.1*
		KL	*qKL10*	*bnlg1185-umc2021*	4.11	0.029	−0.005	A	*9.9*
		KWI	*qKWI6a*	*umc1341-umc1912*	6.71	0.029	−0.006	PD	*17.4*
		KWI	*qKWI7*	*bnlg1305-dupssr11*	5.58	0.021	−0.003	A	*9.3*
		KT	*qKT8a*	*bnlg2082-umc2075*	4.09	0.009	0.005	PD	*6.1*
		KT	*qKT6a*	*umc1444-bnlg249*	3.84	0.016	−0.005	PD	*16.2*
		KV	*qKV10a*	*bnlg1450-bnlg1185*	6.44	0.014	−0.009	PD	*22.3*
		KV	*qKV10c*	*phi323152-umc2351*	5.81	0.013	−0.007	PD	*17.5*
		KV	*qKV6*	*umc1341-umc1912*	4.22	0.011	−0.003	PD	*12*
		KW	*qKW7a*	*umc1987-bnlg1305*	7.46	0.023	0.003	A	*10.6*
		KW	*qKW10a*	*bnlg1185-umc2021*	4.72	0.019	−0.007	PD	*14.9*
		KW	*qKW3a*	*bnlg1160-phi046*	3.96	−0.015	−0.004	PD	*12*
		KW	*qKW7b*	*umc1929-bnlg1808*	3.92	−0.025	0.006	PD	*18*
	Anyang	KL	*qKL3*	*bnlg1647-umc2258*	4.06	−0.04	0.005	A	*11.2*
		KWI	*qKWI6a*	*umc1341-umc1912*	5.56	0.026	−0.006	PD	*15.5*
		KWI	*qKWI2a*	*umc1497-umc2380*	4.71	−0.023	−0.003	A	*11.6*
		KWI	*qKWI7*	*bnlg1305-dupssr11*	4.01	0.018	−0.002	A	*7*
		KT	*qKT6b*	*umc1912-phi452963*	4.49	0.015	−0.006	PD	*21.1*
		KV	*qKV10a*	*bnlg1185-umc2021*	7.25	0.016	−0.013	PD	*26.3*
		KV	*qKV7a*	*bnlg1792-umc1929*	4.72	−0.016	0.006	PD	*17.5*
		KV	*qKV7b*	*umc1401–bnlg1380*	4.21	−0.013	0.002	A	*13.5*
		KV	*qKV7c*	*bnlg1305-dupssr11*	3.96	0.013	−0.006	PD	*15.2*
		KW	*qKW10a*	*bnlg1185-umc2021*	5.49	0.016	−0.004	PD	*16.6*
		KW	*qKW5a*	*umc1524-umc1537*	4.91	−0.017	−0.001	A	*17.3*
2010	Zhengzhou	KL	*qKL5b*	*umc1482-bnlg1847*	4.17	−0.027	−0.011	PD	*6.7*
		KWI	*qKWI6a*	*umc1341-umc1912*	5.62	0.027	−0.008	PD	*17.1*
		KT	*qKT2b*	*umc1185-umc1579*	6.23	0.014	0.001	A	*17.4*
		KV	*qKV10a*	*bnlg1185-umc2021*	5.86	0.017	−0.011	PD	*23.8*
		KV	*qKV10b*	*bnlg1450-bnlg1185*	6.45	0.016	−0.012	PD	*25.6*
		KW	*qKW7a*	*umc1987-bnlg1305*	6.68	0.021	0.002	A	*12.7*
		KW	*qKW7b*	*umc1929-bnlg1808*	4.98	−0.021	0.003	A	*18.9*
		KW	*qKW10b*	*phi323152-umc2351*	4.15	0.013	−0.005	PD	*10.7*
	Anyang	KL	*qKL3*	*bnlg1647-umc2258*	5.69	−0.046	0.016	PD	*16.2*
		KWI	*qKWI6a*	*umc1341-umc1912*	5.55	0.03	−0.007	PD	*18.4*
		KWI	*qKWI7*	*bnlg1305-dupssr11*	3.82	0.019	−0.001	A	*7.7*
		KT	*qKT2a*	*phi109642-umc1185*	5.83	0.015	−0.001	A	*18.2*
		KT	*qKT8b*	*umc1360-umc1872*	5.39	0.014	0.003	A	*14*
		KT	*qKT1*	*bnlg439-umc2390*	4.01	−0.014	0.014	D	*14.7*
		KV	*qKV10a*	*bnlg1185-umc2021*	5.87	0.015	−0.011	PD	*24.9*
		KV	*qKV10b*	*bnlg1450-bnlg1185*	6.45	0.016	−0.012	PD	*22.3*
		KW	*qKW7a*	*umc1987-bnlg1305*	6.54	0.018	0.002	A	*10.5*
		KW	*qKW10a*	*bnlg1185-umc2021*	4.24	0.019	−0.007	PD	*13.5*
Joint across environments		KL	*qKL3*	*bnlg1647-umc2258*	5.49	−0.046	0.007	A	*15.2*
		KWI	*qKWI2b*	*umc1185-umc1579*	4.4	0.011	−0.002	PD	*12.2*
		KWI	*qKWI6b*	*umc1912-phi452693*	4.07	0.014	−0.007	PD	*16.1*
		KWI	*qKWI6c*	*umc1444-bnlg249*	3.52	0.013	−0.004	PD	*12.4*
		KT	*qKT2c*	*umc2402-umc1497*	3.99	-0.023	−0.004	A	*11.2*
		KT	*qKT3*	*bnlg1144-bnlg1647*	3.53	−0.023	0.022	D	*10.4*
		KT	*qKT6c*	*umc1341-umc1912*	6.3	0.026	−0.007	PD	*16.2*
		KT	*qKT7*	*bnlg1305-dupssr11*	4.44	0.019	−0.003	A	*7.5*
		KV	*qKV10a*	*bnlg1185-umc2021*	8.56	0.017	−0.011	PD	*33.2*
		KW	*qKW7a*	*umc1987-bnlg1305*	6.78	1.619	0.231	A	*8.3*
		KW	*qKW7b*	*umc1929-bnlg1808*	3.83	−2.346	0.591	PD	*21.6*
		KW	*qKW10a*	*bnlg1185-umc2021*	5.82	1.723	−0.802	PD	*17.1*

Notes: ^a^ KL, kernel length; KWI, kernel width; KT, kernel thickness; KV, kernel volume; KW, kernel weight;

b QTL, q + trait abbreviation + chromosome number + QTL number, e.g., *qKW7a*,corresponds to the first QTL for KW on chromosome 7;

c Logarithm of odds for each QTL;

d A, additive values (a positive or negative value indicates that the additive effect was derived from Huang C or Xu 178, respectively); D, dominant values;

e Effect of each QTL: A, additive; PD, partial dominance; D, dominance;

f R^2^ contribution.

Based on joint QTL mapping across the four environments, 12 main QTL for the five measured kernel-related traits were detected on chromosomes 2, 3, 6, 7, and 10. *qKL3,* which exhibited a 15.24% phenotypic contribution to KL, was recorded at the Anyang location during both years of the study. Three new QTL for KWI, *qKWI2b*, *qKWI6b*, and *qKWI6c*, were responsible for 12.2%, 16.1%, and 12.4% of the total variance, respectively. Four new QTL for KT were detected, including the *qKT6c* and *qKT7* were located in chromosomal bins 6.04–6.06 and 7.02–7.03, respectively. *qKV10a* was the only one QTL for KV, which displayed a high contribution for 33.2% to the total mean phenotypic variance, and was also detected simultaneously in the four environments. Three common QTL were found for KW among the four environments: *qKW7a*, *qKW7b*, and *qKW10a*. The *qKW7b* locus explained the highest total mean phenotypic variance for 21.58%.

### QTL for KW conditioned on the other four kernel-related traits

Conditional QTL mapping for KW was performed using the phenotypic values of KW conditioned on the other four measured kernel-related traits in every environment. Based on this mapping, 14 conditional main QTL for KW were detected (Table 4; Fig. 1). In 2009, seven conditional QTL were identified at Zhengzhou. Of the four unconditional QTL detected in each corresponding environment, *qKW10a* and *qKW7a* were also identified for KW conditioned on KL (KW|KL). Compared with their corresponding unconditional QTL, the two conditional QTL for KW|KL showed slightly decreased additive effects. When KW was conditioned on KWI, KT, and KV, there were two, two, and zero extra conditional QTL identified for KW, respectively. In 2009 at the Anyang location, two conditional QTL were detected when KW was conditioned on the four kernel traits. Of the two unconditional QTL for KW in 2009 at Anyang, *qKW10a,* was identified for KW|KL, and *qKW5a* was identified for KW|KT. The two conditional QTL showed similar additive effects towards the corresponding unconditional QTL. At Zhengzhou in 2010, two QTL for KW|KL, were also identified by unconditional QTL mapping. The two conditional QTL showed additive effects, which were similar to those of the corresponding unconditional QTL. In addition, one new QTL for KW|KT was detected. At Anyang in 2010, two conditional QTL for KW|KL and KW|KTWI were identified. *qKW10a*, which is a QTL for KW|KL, was also detected by unconditional QTL mapping.

**Table 4:** Conditional QTL for kernel weight conditioned on the four other kernel-related traits and kernel volume conditioned on three kernel-structure characters in the immortalized F_2_ population.

Year	Location	Trait^a^	QTL^b^	Markers interval	LOD^c^	A^d^	D^d^	Effects^e^	R^2^(%)^f^
2009	Zhengzhou	KW|KL	*qKW10a*	*bnlg1185-umc2021*	6.4	0.015	−0.008	PD	*23.4*
		KW|KL	*qKW7a*	*umc1987-bnlg1305*	5.95	0.02	0.003	A	*12.0*
		KW|KL	*qKW5b*	*umc2159-phi331888*	4.02	−0.009	−0.008	D	*5.1*
		KW|KWI	*qKW10c*	*bnlg1450-bnlg1185*	4.26	0.012	−0.003	PD	*16.6*
		KW|KWI	*qKW10b*	*phi323152-umc2351*	4.15	0.012	−0.005	PD	*15.0*
		KW|KT	*qKW3b*	*bnlg1647-umc2258*	5.5	−0.048	0.011	PD	*14.6*
		KW|KT	*qKW4*	*umc1662-bnlg2291*	4.52	0.036	0.003	A	*9.8*
		KV|KL	*qKV2*	*umc1497-umc2380*	4.13	−0.007	−0.008	D	*5.3*
		KV|KL	*qKV10d*	*umc2351-bnlg1450*	6.56	0.013	−0.007	PD	*19.5*
		KV|KWI	*qKV10d*	*umc2351-bnlg1450*	4.81	0.009	−0.003	PD	*14.8*
		KV|KT	*qKV6*	*umc1341-umc1912*	4.56	0.011	−0.002	A	*16.7*
	Anyang	KW|KL	*qKW10a*	*bnlg1185-umc2021*	3.74	0.015	−0.005	PD	*14.5*
		KW|KT	*qKW5a*	*umc1524-umc1537*	3.92	−0.010	−0.011	D	*7.2*
		KV|KL	*qKV10a*	*bnlg1185-umc2021*	8.82	0.020	−0.012	PD	*36.1*
		KV|KWI	*qKV10b*	*bnlg1450-bnlg1185*	5.57	0.012	−0.008	PD	*22.1*
		KV|KT	*qKV10a*	*bnlg1185-umc2021*	5.43	0.015	−0.017	D	*25.5*
2010	Zhengzhou	KW|KL	*qKW7a*	*umc1987-bnlg1305*	6.26	0.019	0.001	A	*12.8*
		KW|KL	*qKW7b*	*umc1929-bnlg1808*	4.78	−0.021	0.004	A	*21.3*
		KW|KT	*qKW3c*	*umc2377-bnlg1523*	4.94	−0.009	0.020	OD	*5.9*
		KV|KL	*qKV10b*	*bnlg1450-bnlg1185*	6.42	0.016	−0.011	PD	*23.8*
		KV|KWI	*qKV10b*	*bnlg1450-bnlg1185*	5.64	0.013	−0.007	PD	*22.2*
		KV|KT	*qKV10a*	*bnlg1185-umc2021*	4.45	0.015	−0.010	PD	*28.4*
	Anyang	KW|KL	*qKW10a*	*bnlg1185-umc2021*	5.06	0.014	−0.012	PD	*23.2*
		KW|KWI	*qKW5c*	*bnlg2305-umc1792*	4.5	−0.001	−0.015	OD	*6.1*
		KV|KL	*qKV10b*	*bnlg1450-bnlg1185*	4.00	0.012	−0.007	PD	*18.0*
		KV|KL	*qKV10a*	*bnlg1185-umc2021*	3.85	0.013	−0.008	PD	*18.6*
		KV|KWI	*qKV2*	*umc1497-umc2380*	4.46	0.010	−0.008	D	*19.0*
Joint Across Environments		KW|KL	*qKW7a*	*umc1987-bnlg1305*	6.12	1.592	0.257	A	*8.8*
		KW|KL	*qKW10a*	*bnlg1185-umc2021*	5.65	1.514	−1.015	PD	*21.3*
		KV|KL	*qKV10b*	*bnlg1450-bnlg1185*	7.89	0.016	−0.011	PD	*31.3*
		KV|KL	*qKV7c*	*bnlg1305-dupssr11*	4.03	0.013	−0.006	PD	*17.6*
		KV|KWI	*qKV10a*	*bnlg1185-umc2021*	4.98	0.015	−0.011	PD	*26.9*
		KV|KT	*qKV10b*	*bnlg1450-bnlg1185*	5.80	0.010	−0.006	PD	*21.8*

Notes: ^a^ KW, kernel weight; KW|KL, kernel weight conditioned on kernel length; KW|KWI, kernel weight conditioned on kernel width; KW|KT, kernel weight conditioned on kernel thickness; KW|KV, kernel weight conditioned on kernel volume;

b QTL, q + trait abbreviation + chromosome number + QTL number, e.g., *qKW7a*,corresponds to the first QTL for KW on chromosome 7;

c Logarithm of odds for each QTL;

d A, additive values (positive or negative values indicate that the additive effect was derived from Huang C or Xu 178, respectively); D, dominant values;

e Effect of each QTL: A, additive; PD, partial dominance; D, dominance; OD, overdominance;

f R^2^ contribution.

The mean values of the five measured kernel-related traits in the four environments were used to calculate conditional values for the joint conditional QTL mapping of KW. Two of the three unconditional QTL for KW, *qKW7a* and *qKW10a*, were identified for KW|KL(Table 4). Compared with the corresponding unconditional QTL, the two conditional QTL showed additive effects similar to their corresponding unconditional QTL. No QTL for KW|KWI, KW|KT, or KW|KV were identified throughout the analysis.

### Conditional QTL mapping for KV conditioned on kernel-shape traits

Conditional QTL mapping of KV on the three kernel-shape traits, KL, KWI, and KT, resulted in 17 conditional main QTL for KV distributed on four chromosomes (Table 4; Fig. 1). In the 2009 experiments, four such QTL were identified at Zhengzhou. One QTL for KV|KT, *qKV6*, was also detected using unconditional QTL mapping, and showed a slightly reduced additive effect compared to the corresponding unconditional QTL. For KV|KL and KV|KWI, there were two and one new QTL detected, respectively. At Anyang in 2009, three conditional QTL were identified when KV was conditioned on the three kernel-shape traits. Of the three conditional QTL, *qKV10b*, which was identified for KV|KWI was also identified in the same environment by unconditional QTL mapping. With respect to the four unconditional QTL detected for KV in the corresponding environments, four, three and four were undetectable when KV was conditioned on KL, KWI and KT, respectively. At Zhengzhou in 2010, three QTL for KV conditioned on the three kernel-shape traits were also detected by unconditional mapping: *qKV10b*, which was detected for both KV|KT and KV|KL, and *qKV10a* identified for KV|KWI. These three conditional QTL all showed additive effects similar to their corresponding unconditional QTL. At Anyang, three conditional QTL for KV were identified, two of which were detected for KV|KL and that were also by unconditional QTL mapping. Compared with the corresponding unconditional QTL, the two conditional QTL both showed smaller additive effects in the conditional mapping analysis.

Using joint conditional QTL mapping for KV, four QTL were detected. The only unconditional QTL, *qKV10b*, was undetectable when KV was conditioned on KWI. The conditional QTL identified for KV|KL and KV|KT showed similar additive effects to the unconditional ones.

### Detection of digenic epistatic effects for measured kernel-related traits

Digenic epistatic effects involving the five measured kernel-related traits were identified using the QTLNetwork 2.1 software package [35]. A total of 18 pairs of epistatic interactions were detected (Table 5, 6). These interacting pairs were associated with 34 loci on all ten chromosomes. Strikingly, only a small proportion of the identified epistatic loci coincided with the main-effect QTL detected by unconditional and conditional QTL mapping, which include *qKL3*, *qKL10*, *qKWI6a*, *qKV7c*, *qKV10a*, and *qKW10c*. Of the 18 interactions, twelve and nine of these were determined with significant AA and DD epistatic effects, accounting for 67% and 50% of all epistatic interactions, respectively. For KL and KT, the AA interactions exhibited large epistatic effects, one interaction contributing 4.6% to KL phenotypic variance, and two interactions contributing for 4.3% and 3.5% of KT phenotypic variance. According to KL and KW, DD interactions contributed large epistatic effects, with 7–8/10–15 exhibiting a high contribution of 6.7% towards KL phenotypic variance. Additionally, a DA interaction was identified to account high epistatic effects, which accounted for 4.0% of KWI phenotypic variance.

**Table 5:** Digenic epistatic effects detected for the five kernel-related traits in the immortalized F_2_ population.

Trait	Chr-ini^a^	Marker interval	Chr-inj^a^	Marker interval	AiAj^b^	R^2^(%)^c^	AiDj^b^	R^2^(%)	DiAj^b^	R^2^(%)	DiDj^b^	R^2^(%)
KL	3–9	***bnlg1647-umc2258*** e	4–12	*umc1194-umc1847*							−0.045^**^	*1.2*
	4–9	*umc1662-bnlg2291*	8–1	*bnlg1056-umc2052*	0.037^**d^	*3.0*			−0.025^*^	*0.1*	0.049^**^	*1.3*
	1–18	*umc1955-phi038*	10–19	***bnlg1185-umc2021***	0.042^**^	*4.6*					0.056^**^	*1.1*
	2–18	*bnlg1520-phi101049*	8–15	*umc2075-umc1360*	−0.032^**^	*2.5*					0.043^**^	*1.6*
	7–8	*bnlg1808-umc1987*	10–15	*umc2172-phi323152*							−0.090^**^	*6.7*
KWI	2–5	*bnlg1909-umc1003*	6-3	***umc1341-umc1912***	0.019^**^	*2.7*						
	5–7	*bnlg1879-umc1557*	6–11	*umc1257-umc1083*	0.018^**^	*1.7*						
	6–24	*phi423796-bnlg1600*	10–10	*umc2122-umc1993*	−0.021^**^	*2.9*			0.032^**^	*4.0*		
KT	1–13	*umc2025-umc1988*	8–7	*mmc0181-bnlg1812*	−0.012^**^	*4.3*						
	1–14	*umc1988-umc1396*	8–5	*umc2031-bnlg240*							−0.016^**^	*2.0*
	2–9	*umc2402-umc1497*	4–9	*umc1662-bnlg2291*					−0.020^**^	*3.2*		
	5–9	*umc2159-phi331888*	10–1	*phi118-umc1291*	−0.013^**^	*3.5*						
KV	1–17	*umc1245-umc1955*	10–18	***bnlg1450-bnlg1185***	0.007^**^	*2.6*					−0.016^**^	*1.1*
	7–10	***bnlg1305-dupssr11***	10–18	***bnlg1450-bnlg1185***			−0.014^**^	*1.6*				
	2–8	*bnlg1036-umc2402*	9–2	*bnlg1724-umc1430*			0.008^*^	*0.9*				
KW	3–8	*bnlg1144-bnlg1647*	5–16	*bnlg1847-umc1524*	0.012^**^	*1.2*					−0.025^**^	*2.1*
	3–14	*umc1773-umc1495*	10–18	***bnlg1450-bnlg1185***	0.010^**^	*1.7*						
	3–22	*bnlg1160-phi046*	10–7	umc2350-umc1697	−0.010^**^	*0.8*	−0.013^**^	*1.2*			0.027^**^	*2.8*

Notes:^ a^ Chr-ini is the first marker interval chromosomal location, and Chr-inj is the second marker interval chromosomal location;

b The digenic effect of the two interacting loci; AiAj, the effect of additive-by-additive interaction between points i and j; AiDj, the effect of additive-by-dominant interaction between points i and j; DiAj, the effect of dominant-by-additive interaction between points i and j; DiDj, the effect of dominant-by-dominant interaction between points i and j; a positive or negative epistatic effect indicates that parental allele or recombinant allele combinations, respectively, increase phenotypic values;

increase phenotypic values;

c Contribution explained by the locus pair interaction;

d * *p*≤0.0005; ^**^ *p*≤0.0001;

e Bold indicates that the interval is identical to conditional or unconditional QTL.

**Table 6:** Summary of the digenic epistatic analysis for the five kernel-related traits in the immortalized F_2_ population.

Trait	Interactions/Loci ^a^	Chromosomes b	AA		AD/DA		DD	
			Number c	R^2^ (%) d	Number c	R^2^ (%) d	Number c	R^2^ (%) d
KL	5/10	7	3	*2.5*–*4.6*	1	*0.1*	5	*1.1*–*6.7*
KWI	3/6	4	3	*1.7*–*2.9*	1	*4.0*		
KT	4/8	6	2	*3.5*–*4.3*	1	*3.2*	1	*2.0*
KV	3/5	5	1	*2.6*	2	*0.9*–*1.6*	1	*1.1*
KW	3/6	3	3	*0.8*–*1.7*	1	*1.2*	2	*2.1*–*2.8*
Total	18/34	10	12		6		9	

Notes: ^a^ The number of epistasis interactions/loci involved;

b The number of chromosomes the loci were distributed upon;

c The number of the corresponding epistatic interactions;

d Contribution explained by the locus pair interaction.

### Detection of conditional digenic epistatic effects for KW and KV

Digenic epistatic analysis for KW conditioned on the other four kernel-related traits and KV conditioned on the three kernel shape characters identified 13 and 9 conditional digenic interactions, which involved 25 and 16 loci, respectively (Table 7). All the conditional digenic interactions were identified as new interactions that in addition to the unconditional digenic interactions detected for KW and KV (i. e. 9–15/10–18 and 2–8/6–2). Among the loci involved in conditional KW digenic epistasis, *qKW10c* and *qKW7b*, were identified by unconditional or conditional QTL mapping at loci 10–18 and 7–7, respectively. However, no conditional epistatic locus for KV was consistent with the main QTL identified for KV. Of the thirteen epistatic interactions identified for KW conditioned on the other four measured kernel-related traits, nine and eight interactions were identified with significant AA and DD effects, accounting for 69.2% and 61.5% of all interactions, respectively. In contrast, four, six, four and five interactions were identified with significant AA, AD, DA and DD effects in the nine conditional epistatic interactions for KV on the three kernel-shape traits, accounting for 44.4%, 66.7%, 44.4% and 55.6% of all interactions, respectively.

**Table 7:** Digenic epistatic effects detected for kernel weight conditioned on four other kernel-related traits and kernel volume conditioned on three kernel-structure characteristics in the immortalized F_2_ population.

Trait	Chr-ini^a^	Marker interval	Chr-inj^a^	Marker interval	AiAj^b^	R^2^(%)^c^	AiDj^b^	R^2^(%)	DiAj^b^	R^2^(%)	DiDj^b^	R^2^(%)
KW|KL	9–15	*umc1714-umc1982*	10–18	***bnlg1450-bnlg1185***	−0.011^**^	*3.6*						
	2–11	*umc2380-bnlg1329*	5–14	*umc1722-umc1482*	0.008^**^	*2.1*					0.023^**^	*0.6*
	2–16	*bnlg2144-umc1464*	7–7	***umc1929-bnlg1808***			0.014^**^	*1*			−0.013^*^	*0.9*
	2–17	*umc1464-bnlg1520*	5–9	*umc2159-phi331888*							−0.028^**^	*3.1*
	2–18	*bnlg1520-phi101049*	7–5	*umc1666-bnlg1792*	0.008^**^	*1.2*	−0.019^**^	*5.1*			−0.014^*^	*0.9*
	4–11	*bnlg1784-umc1194*	9–3	*umc1430-umc1037*	0.009^**^	*2.2*						
KW|KWI	3–13	*umc1174-umc1773*	10–13	*bnlg1677-umc1640*			−0.012^**^	*2.5*				
KW|KT	1–26	*phi227562-bnlg504*	3–15	*umc1495-umc2261*			0.013^**^	*0.4*			0.046^**^	*7.1*
	3–1	*umc1793-phi104127*	10–4	*umc1432-bnlg1716*	−0.013^**^	*1.6*			0.015^**^	*1.9*		
	6–25	*bnlg1600-umc2196*	10–15	*umc2172-phi323152*	−0.009^**^	*1.4*					0.033^**^	*6.6*
KW|KV	1–7	*umc1044-phi339017*	4–1	*phi072-umc1017*	0.013^**^	*8.8*			−0.015^**^	*1.7*	0.024^**^	*3.5*
	1–23	*phi308707-umc1553*	4–4	*umc1288-umc2280*	0.007^**^	*1.5*					−0.027^**^	*5.5*
	1–23	*phi308707-umc1553*	4–6	*umc1945-umc1963*			0.007^*^	*0.6*				
KV|KL	2–8	*bnlg1036-umc2402*	6–2	*phi299852-umc1341*			−0.025^**^	*5.4*			0.028^**^	*2.7*
	2–18	*bnlg1520-phi101049*	6–19	*bnlg1867-umc1444*	−0.01^**^	*2.5*	0.010^**^	*1.5*			0.025^**^	*4.2*
	4–6	*umc1945-umc1963*	7–9	*umc1987-bnlg1305*	0.005^*^	*0.8*	−0.009^**^	*3.7*				
KV|KWI	1–4	*bnlg1429-bnlg1803*	10–9	*umc2043-umc2122*					−0.021^**^	*2.8*		
	4–7	*umc1963-umc1652*	4–9	*umc1662-bnlg2291*			−0.010^**^	*0.6*	0.014^**^	*5*	0.016^**^	*1.4*
	4–7	*umc1963-umc1652*	7–1	*mmc0171-umc1545*					−0.007^**^	*2.1*	−0.012^**^	*1.9*
KV|KT	3–22	*bnlg1160-phi046*	7–11	*dupssr11-dupssr9*	−0.014^**^	*4.3*					0.021^**^	*1.5*
	6–14	*umc1006-bnlg1188*	7–2	*umc1545-umc1401*	0.007^*^	*0.03*	0.011^**^	*0.1*				
	6–18	*umc2312-bnlg1867*	7–2	*umc1545-umc1401*			−0.021^**^	*2*	−0.011^**^	*0.3*		

Notes:^ a^ Chr-ini is the first marker interval chromosomal location, and Chr-inj is the second marker interval chromosomal location;

b The digenic effect of the two interacting loci; AiAj, the effect of additive-by-additive interaction between points i and j; AiDj, the effect of additive-by-dominant interaction between points i and j; DiAj, the effect of dominant-by-additive interaction between points i and j; DiDj, the effect of dominant-by-dominant interaction between points i and j; a positive or negative epistatic effect indicates that the parental allele or recombinant allele combinations, respectively, increase phenotypic values;

c Contribution explained by the locus pair interaction;

d * *p*≤0.0005; ^**^ *p*≤0.0001;

e Bold indicates the interval is identical to conditional or unconditional QTL.

## Discussion

Of the 54 unconditional main QTL identified for five kernel-related traits, many of which clustered in chromosomal bins 10.06–10.07, 6.04–6.06, and 7.02–7.03. In chromosomal bin 10.06–10.07, the QTL for all the five kernel-related traits were all clustered together. The common QTL, *qKW10a*, *qKV10a*, and *qKV10b*, detected in multiple environments and joint QTL mapping across environments, were largely responsible for the phenotypic variance of their corresponding traits. Within the 10.06–10.07 genomic region, Peng et al. (2011) identified QTL for KW, KV, KL, and KWI in two F_2∶3_ populations [8]; and also within this region, a QTL for grain yield was found by Tuberosa et al. (2002) [36]. These observations indicate that this particular genomic region seems to be very important for kernel development and grain yield. A common QTL across multiple environments, *qKWI6a*, identified in 6.04–6.06 chromosomal bin, was clustered with QTL for both KV and KT. In our previous study, using the same IF_2_ population, a QTL was located for maize grain-filling rate in chromosomal bin 6.05–6.06 [37]. We predict that for the 6.04–6.06 chromosomal bin, there exists important genes for kernel development, but there is likely specific expression of these genes depending on the genetic background. In the genomic region 7.02–7.03, *qKW7a* was identified within a cluster containing *qKW7b* and *qKWI7*. Interestingly, Peng et al. (2011) also identified important QTL for KW and KWI in the 7.02 bin using a F_2∶3_ population [8]. Goldman et al. (1993) identified a QTL for 300-kernel weight in the 7.02–7.03 region using Illinois long term selection maize strains [38]. Additionally, *qKL3* was identified in the 3.02–3.03 chromosomal bin in multiple environments and via joint QTL mapping. Within the same chromosomal bin, a QTL for heterosis of ear length was identified in an IF_2_ population [39], a QTL for rate of kernel production was recorded in an F_2∶3_ population [22], and a QTL for KT was detected in an F_2∶3_ population [8]. These reports suggest that this genomic region is very important for grain-yield under different genetic backgrounds because of the presence of yield-related genes.

Epistasis, an important genetic phenomenon underlying quantitative trait variation, has been shown to exert large effects on heterosis and grain yield in maize [17], [18], [39]. In our study, of the18 pairs of interactions for the five kernel-related traits were identified, 67% and 50% of them showed significant AA and DD effects. AA interactions displayed the highest proportion out of the four types of epistatic interactions, a same trend identified in previous studies in maize [17], [39]. In rice, Hua et al. (2002) also found that AA interactions accounted for a larger proportion compared to AD, DA, and DD in an IF_2_ population [34]. In the present study, the large effects from DD interactions were likely specific in to the genetic background and population used. The corresponding epistasis interaction showed large AA and DD effects on KL and KT, which revealed that AA and DD epistatic interactions are important genetic and heterosis components for KL and KT in maize but have little effect on other kernel-related traits. This result was consistent with the KL and KT phenotypic performance. For instance, KL of the hybrid (Nongda 108) and IF_2_ population showed high heterosis, whereas the KT values of these hybrid crosses (Nongda 108 and the IF_2_ population) were smaller than their parents (Huang C and Xu 178) seriously (Table 1). Additionally, only a small proportion of loci involved in epistasis interactions were coupled with the determined unconditional main-effect QTL, which is consistent with previous studies [8], [17], [18], [39].

Conditional QTL mapping provides an efficient tool for dissecting the genetic interrelationship between KW or KV and their components for either trait at the individual QTL level. Of the 14 unconditional QTL determined for KW, eight of them were independent of KL, while one QTL was partially determined by KT. Across the four environments and joint environments, five, zero, one, one and zero additional conditional QTL, were identified for KW by excluding the influence of related kernel traits. These results suggest that KWI and KV, followed by KT, had the strongest influence on KW, whereas KL contributed the least. Through examination of these kernel-related traits in two F_2∶3_ maize populations, Peng et al. (2011) demonstrated that KW is genetically correlated highly with KV, KT, and KWI [8]. Of the 12 unconditional QTL for KV in the four environments and joint environments, two, zero and two QTL were independent of KL, KWI and KT, respectively; two, two and one QTL were partial contributions from KL, KWI and KT, respectively; and eight, ten and nine QTL were entirely determined by KL, KWI, and KT, respectively. In conclusion, KT, followed by KWI contributed the strongest influence on KV, while KL had the weakest influence.

For the genetic interrelationships between two associated traits, there are no previous studies conducted at the digenic epistatic level. In the analysis of our study, none of the three unconditional digenic interactions for KW were identified during the conditional digenic epistatic analysis. Conditional epistatic interactions were identified by excluding the influence of other kernel parameters, and we identified all were new interactions. These results revealed that epistatic interactions for KW were entirely contributed by the four other kernel-related traits. A similar conclusion can also be drawn for the relationships between KV and the three kernel shape characteristics (i.e. KV|KL, KV|KWI and KV|KT) that epistatic interactions for KV were entirely dependent on the contribution of the three kernel shape characters. Compared to the conditional epistatic interactions identified for KW and KV, in all the four types of interactions the proportion of significant AA and DD interactions account for the different trends observed. For KW, AA and DD interactions accounted for a greater proportion of all epistatic interactions compared to AD and DA interactions. In contrast for KV, the AD interaction accounted for the greatest proportion. This difference might be a causative factor in heterosis: the hybrid Nongda 108 and IF_2_ crosses displayed heterobeltiosis in KW, and mid-parent heterosis in KV (Table 1).

Grain yield is a complex trait controlled by multiple genes. During the breeding process, the complexity of genetic control for grain yield increases the difficulty of direct selection. In breeding practice, a perfect kernel structure usually results in a high grain yield [8]. Breeders usually improve KW by selecting for KL since selection for KWI or KT might decrease the number of ear rows or ear kernels. Our results revealed that KWI and KV, followed by KT, had the stronger influence on KW, whereas KL had the weakest influence. This effect suggests that selecting for KL to improve KW is not a valid option. The common QTL detected in multiple environments or the multiple traits identified in this study cannot be used directly in MAS for maize breeding. Among the unconditional QTL identified for KW or KV, several QTL were strongly associated with KL and may be used in marker-assisted selection to improve KW, so as to achieve a perfect kernel structure, thereby reliably improving grain yield.

## Materials and Methods

### Development of the immortalized F_2_ population

A set of 166 RILs was constructed from two elite inbred lines, Huang C and Xu 178 [15], using the single-seed descent method. The elite hybrid of these two lines, Nongda 108, covered approximately 2.7 million hectares in China from 2001 to 2004. An IF_2_ population, comprised of 249 single crosses, was generated via three inter-mating rounds of the 166 RILs following the procedure described by Hua et al. (2002) [34]. Because insufficient seeds were harvested from six of these crosses, only 243 lines were adopted for this study.

### Field evaluation

Trials took place at two locations over two years. Plant materials, including the IF_2_ population, the two parents, and the hybrid, were planted at the agronomy farms of Henan Agricultural University (Zhengzhou, 113°42′E, 34°48′N) in central China (average daily temperature, 14.3°C; average annual rainfall in 2009 and 2010, 640.9 mm) and Anyang Agricultural Institute (Anyang, 114°21′E, 36°6′N) in the center of the North China Plain (average temperature, 14.1°C; average annual rainfall, 556.9 mm). Seeds were planted on 12 June 2009 and 8 June 2010 at Zhengzhou, and on 17 June 2009 and 12 June 2010 at Anyang. The field experiments followed a randomized complete block design with three replicates at each location. Each block was comprised of 6 m long×0.67 m wide rows of 25 plants at a density of 65,250 plants ha^−1^. The fields were kept free of weeds and pests, and irrigated and fertilized to avoid water or nutritional stresses.

### Sampling and measurement of kernel-related traits

Five ears from each plot were hand-collected at physiological maturity and dried completely. Phenotypic data were recorded as follows: (1) KL (cm kernel^−1^)  =  (ear diameter – cob diameter) / 2, where ear and cob diameters were measured at the middle of the ear [7]; (2) kernel width in the middle of a kernel (KWI, cm kernel^−1^)  =  [cob diameter + (ear diameter – diameter of cob) / 2] π / (ear row number) [7]; (3) KT (cm kernel^−1^), estimated from the thickness of 10 kernels in the middle of an ear [7], [8]; (4) KW (g kernel^−1^), the average of three measurements of the weight of 100 randomly-selected kernels; and (5) KV (ml kernel^−1^), calculated from the volume of the 300 weighed kernels. Each phenotypic character was measured five times to evaluate KL and KW. Data analysis was performed using the PROC MIXED procedure of SAS 9.2 [40].

### Unconditional and conditional QTL mapping

A genetic linkage map for the RIL population from which the IF_2_ population was derived was constructed using 217 SSR markers with Mapmaker 3.0 [29]. The map included 10 linkage groups spanning a total of 2438.2 cM, with an average interval of 11.2 cM [15]. The genotypes of each IF_2_ cross were deduced from the marker genotypes of their RIL parents, with QTL mapping in the IF_2_ population performed using the molecular linkage map of the RIL population [34], [39].

Unconditional QTL mapping was performed using the composite interval mapping (CIM) method and Model 6 of the Zmapqtl module of Windows QTL Cartographer 2.5 (Simple F_2_ model) [29]. The logarithm of odds threshold was calculated using 1,000 permutations at a significance level of *p* = 0.05, with scanning intervals of 2 cM between markers and a putative QTL, and a 10 cM window. Background control of marker cofactors was set using five controlling markers by forward-backward stepwise regression. Average values of two replicates for these five kernel-related traits in each environment were used as input data for single environment QTL mapping, and the phenotypic average values of the four environments were calculated to carry out QTL mapping jointly across environments.

Conditional phenotypic values, y_hk_ (T_1_|T_2_), were obtained using a mixed model approach for conditional analysis of quantitative traits as described by Zhu (1995) [2]. The notation T_1_|T_2_ corresponds to trait 1 conditioned on trait 2; for example, KW|KL symbolizes KW conditioned on KL [30]. In this study, we estimated the following conditional phenotypic values: KW|KL, KW|KWI, KW|KT, KW|KV, KV|KL, KV|KWI, and KV|KT. Single environment conditional QTL mapping was performed with values of KW, KV, and the conditional phenotypes in the four environments (two sites × 2 years); the average value of KW, KV, and the conditional phenotypes across four environments were used to perform QTL mapping jointly across environments. Mapping was carried out using the CIM method in Windows QTL Cartographer 2.5 as described for unconditional QTL mapping.

### Detection of digenic interactions

Digenic interactions were analyzed for kernel-related traits using Mixed Composite Interval Mapping (MCIM) as implemented in QTL Network 2.1 (Simple F_2_ model) [35] at significance levels of *p*≤0.0005 and *p*≤0.0001. Each significant digenic interaction was partitioned into four types: the additive effect at both loci (AA); the additive effect at the first locus and dominant effect at the second (AD); the dominant effect at the first locus and additive effect at the second locus (DA); and the dominant effect at both loci (DD) [41]. Conditional digenic epistatic analysis was performed with the same methodology using the conditional phenotypic values of KW and KV.
